# Supplementary material for: Correlates of older adult inpatients’ personal care provision to people with functional difficulties in Ghana
Source: PLoS One. 2020 Oct 2;15(10):e0238693. doi: 10.1371/journal.pone.0238693 (PMC7531847; doi:10.1371/journal.pone.0238693)
Supplement: S1 Appendix — (DOCX) [file pone.0238693.s001.docx]

You are kindly invited to take part in the survey exploring the disability as well as availability of care and support for older people. This study is entirely academic and therefore your responses will only be used for academic purposes. Your response will be treated confidential and will not be revealed to a third party. Your participation in this study will be highly valued.

**Section [1]: Socio-Demographic Characteristics**

**1. Date of Birth** ……………………….…………… 2. **Sex** male female

**3. Marital status** never married currently married / cohabiting separated / divorced widowed

**4. Highest level of education completed**

no education less than primary school primary school Secondary/high school

college/pre-University postgraduate degree

**5. Ethnic background** ………………………………………………………………………………………………………….

**6. Religious denomination**

None Christianity (including roman-catholic, protestant, orthodox, other)

Islam Traditional religion Other …………………………………………………….

**7. Area of residence** Rural Urban

**8. Living arrangements**

Alone With husband/wife With children With husband/wife and children

In extended family house Other ………….………………………

**9. Employment status** Currently working Currently not working

**Section [2]: Support and Care Provided by Older People**

At this point, I will want you to tell me about what you normally do to help your family.

1. **Do you regularly provide care for grandchildren or other people’s children? (*Mark one only)***

1Yes, daily 2 Yes, weekly 3 Yes, occasionally 4 No, never

1. **Do you regularly provide care or assistance (e.g. Personal care, transport) to any other person because of their long-term illness, disability or frailty? (Mark all that apply to you)**

1 Yes, for someone who lives with me 2 Yes, for someone who lives elsewhere

3 No, I do not provide care (go to …q6. if you do not provide care)

1. **How many people with a long-term illness, disability or frailty do you regularly provide care for?**

1 One person 2 Two people 3 More than two people

1. **How often do you provide this care or assistance? (in total, for all the people) mark one only)**

1 Every day, several times a week 2 Once a week, once every few weeks 3 Less often

1. **How much time do you usually spend providing such care or support on each occasion?** (***Mark one only***)

1 All day and night 2 all night 3 all day 4 several hours 5 about an hour
