# Supplementary material for: Correlates of older adult inpatients’ personal care provision to people with functional difficulties in Ghana
Source: PLoS One. 2020 Oct 2;15(10):e0238693. doi: 10.1371/journal.pone.0238693 (PMC7531847; doi:10.1371/journal.pone.0238693)
Supplement: Attachment — Submitted filename: Response to Reviewers1.docx [file pone.0238693.s003.docx]

**Dear Dr Ellen Idler,**

We are very grateful for the privilege given us to revise the manuscript. In this letter, we have responded to the reviewers’ and your comments.

**Response to reviewers**

**Response to reviewers:** “Correlates of older adult inpatients’ personal care provision to people with functional difficulties in Ghana”. Manuscript ID: PONE-D-19-28535

**The author has addressed most of the suggestions for revision made in the two reviews. There are a few remaining issues, however, that can be addressed in a Minor Revision.**

*Authors appreciate the additional Editor’s comments and acknowledgement on the authors side of addressing the comments. We have therefore responded to the reviews comments thoroughly.*

**1. Please reframe the research question in the Abstract and elsewhere. As stated "The purpose of the research is to determine the extent of care provision and particularly examine the socio-demographic correlates of personal care provision by older adults in Ghana" -- this research question is posed as descriptive of the extent of personal care provision by ALL older adults in Ghana (the population-level) but the data come from a highly selected sample of patients, from which no generalizations can be made to any larger group.**

*Thank you for raising this important comments. We have modified in the “abstract” that “The purpose of the* ***research*** *was to examine the personal care provision and socio-demographic* ***correlates*** *of personal care provision by older adults inpatients in Ghana”. This is relevant because the participants were recruited from a hospital setting. They gave their account of how they provide care before they became hospitalised. These same change has been reflected in the Title.*

**2. Please provide a more realistic assessment of the extent of care provision. Only 28% of the sample provided care, which is less than in other studies mentioned in the literature review. The conclusion, that "The findings demonstrate that older adults are resources to the community, mostly towards family members and therefore, should be valued as such" would appear to overstate the findings.**

*Thanks to the reviewer for identifying this important issue. The reviewer should be aware that we measured the extent of care provision by using three questions (Number of people you regularly care, Often assistance is provided, and Number of times spent on care –see Table 3). These three questions were asked to explore participant’s initial response to the dependent variable question “Do you regularly provide care or assistance (e.g. bathing, transport) to any other person because of their long-term illness, disability or frailty before you were hospitalised?” Authors have also altered this by providing in the “conclusion” that “Though a small proportion of older adults receiving health care from the hospital setting provide personal care, the findings in this study demonstrate that older adults in the community may be resourceful to the community and their family members due to their relatively better health. Moreover, the study’s findings draw attention to the need to provide financial and health support to older adults receiving healthcare who do not provide personal care to enable them be productive.”*

*Moreover, the authors have specified the nature of participants as “older adults inpatients” to differentiate participants from the general population.*

**3. The information in Table 1 is repeated in Table 2, thus Table 1 is not necessary.**

*Thank you for this suggestion. Table 1 specifies the percentages of the individual categories the respective variables. However, in Table 2 it specifies the row total of the variables in relation to personal care. These are two different tables and due to this, the authors think that both Tables should be maintained.*

**4. Table 2 has several typos (missing parentheses, stray decimal points) -- please correct.**

*Thanks for this emphasis. Authors have addressed all errors in Table 2.*

**5. Table 4 has rows that do not line up.**

*Thanks to the reviewer for drawing attention to this issue. I have corrected the problem in the Table 4.*

**6. Please attend more closely to editing the manuscript. Reviewer 2 points out some grammatical or usage errors.**

*Thanks very much. We have edited the manuscript for grammatical errors. Thanks*
